# Microbiome analysis in Lascaux Cave in relation to black stain alterations of rock surfaces and collembola

**DOI:** 10.1111/1758-2229.13133

**Published:** 2022-11-24

**Authors:** Lise Alonso, Thomas Pommier, Laurent Simon, Flavien Maucourt, Jeanne Doré, Audrey Dubost, Van Trân Van, Guillaume Minard, Claire Valiente Moro, Christophe J. Douady, Yvan Moënne‐Loccoz

**Affiliations:** ^1^ Univ Lyon, Université Claude Bernard Lyon 1, CNRS, INRAE, VetAgro Sup, UMR5557 Ecologie Microbienne Villeurbanne France; ^2^ Univ Lyon, Université Claude Bernard Lyon 1, CNRS, ENTPE, UMR5023 LEHNA Villeurbanne France

## Abstract

Anthropization of Palaeolithic caves open for tourism may favour collembola invasion and result in the formation of black stains attributed to pigmented fungi. However, ecological processes underpinning black stain formation are not fully understood. Here, we tested the hypotheses that black stains from the Apse room of Lascaux Cave display a specific microbiota enriched in pigmented fungi, and that collembola thriving on the stains have the potential to consume and disseminate these black fungi. Metabarcoding showed that the microbiota of black stains and neighbouring unstained parts strongly differed, with in black stains a higher prevalence of *Ochroconis* and other pigmented fungi and the strong regression of *Pseudomonas* bacteria (whose isolates inhibited in vitro the growth of pigmented fungi). Isotopic analyses indicated that *Folsomia candida* collembola thriving on stains could feed on black stain in situ and assimilate the pigmented fungi they were fed with in vitro. They could carry these fungi and disseminate them when tested with complex black stains from Lascaux. This shows that black stain formation is linked to the development of pigmented fungi, which coincides with the elimination of antagonistic pseudomonads, and points towards a key role of *F. candida* collembola in the dynamics of pigmented fungi.

## INTRODUCTION

Dissolution of limestone bedrocks by water leads to the formation of karstic caves (Cuezva et al., 2009). In the past 180,000 years or so, hundreds of caves (if not more) have been used by humans as shelters or to develop parietal art (Jaubert et al., 2016). During the last century, many of these Palaeolithic caves (i.e. caves with Palaeolithic artwork) have been opened for tourist visits (Alonso et al., 2019; Bastian et al., 2009; Bontemps et al., 2021). However, tour operations can change environmental conditions in caves, as a consequence of transformations (e.g. light systems) meant to assist visits, human physiology and behaviour, and the introduction of allochthonous microorganisms and arthropods especially collembola (Alonso et al., 2019; Bastian et al., 2009, 2010). These anthropic effects can stimulate the proliferation of certain microorganisms and lead to alteration of prehistoric art, and several of these caves had to be closed to the public, in particular the UNESCO caves Altamira (Spain) and Lascaux (France).

Among Palaeolithic caves, it is at Lascaux that anthropization went the farthest (Bontemps et al., 2021). Lascaux Cave, discovered in 1940, received more than 1 million visitors from 1948 to 1963. Several alterations developed successively in the cave, and they prompted specific microbial monitoring (Alonso et al., 2018, 2019; Bastian et al., 2010; Martin‐Sanchez, Nováková, et al., 2012a; Saiz‐Jimenez, 2012). In particular, black stains (termed ‘black disease’) appeared on walls late 2001 and especially from 2006 on (Alonso et al., 2018; Bastian et al., 2010; Bontemps et al., 2021; De la Rosa et al., 2017; Martin‐Sanchez, Nováková, et al., 2012a; Saiz‐Jimenez, 2012). These black stains (Figure S1) represent the ultimate stage of surface deterioration resulting from anthropization of Palaeolithic and other show caves (Alonso et al., 2018, 2019; Bastian et al., 2009, 2010; De la Rosa et al., 2017; Martin‐Sanchez et al., 2013; Martin‐Sanchez, Nováková, et al., 2012a).

Collembola with *Folsomia candida* morphology have been evidenced in various stained parts of Lascaux Cave, almost exclusively on black stains (Figure S1) rather than unstained neighbouring parts (Bastian et al., 2010). Collembola from a Slovakian cave and raised on plates where black fungi were grown displayed gut darkening, suggesting fungal consumption (Bastian et al., 2010), and fungal conidia were present in their faeces (Bastian et al., 2010). However, these data were obtained in vitro. Whether microbiome alterations take place in black stains is unknown, the ability of collembola to use black fungi as significant carbon source is not established, and whether a relation exists between collembola‐associated microorganisms and black stain microbiota remains obscure.

Black stain alterations represent a key concern in Palaeolithic caves that have undergone tourism‐related anthropization (Bontemps et al., 2021). Here, we used Lascaux to test the hypotheses initiated in previous studies (Bastian et al., 2010; Martin‐Sanchez, Sanchez‐Cortes, et al., 2012) that a specific microbial community could establish in black stains resulting from cave anthropization, and that collembola thriving on black stains (Figure S1) had the potential to consume and disseminate black fungi present within stains. Illumina MiSeq sequencing was used to characterize microbiome modifications in black stains, and we clarified the ability of collembola to trigger stain formation by selective predation of cave wall microorganisms and spread of black fungi.

## EXPERIMENTAL PROCEDURES

### Sampling

Lascaux Cave is located near Montignac in Périgord (South‐West France). The Apse in Lascaux Cave was selected for sampling due to the presence of black stains with recent growth. We carried out five sampling campaigns, in June 2015 (MiSeq, isotopes), June 2016 (MiSeq), December 2016 (MiSeq), February 2017 (*Pseudomonas* isolation) and May 2017 (additional collembola for MiSeq), using several areas selected on the left and right walls of the room (left and right considered from the cave entrance). Due to Lascaux management rules related to cave vulnerability, sampling was distributed over several days to limit the duration of human presence on a given day.

Collembola and black stains on which they were present were sampled for metabarcoding characterization of associated microorganisms, *Pseudomonas* isolation (from wall samples), and isotopic analysis. To this end, collembola were collected (17 black stain samples, making 710 individuals in total) using sterile insect mouth aspirators (Rose Entomology, Benson, AZ). Wall samples were collected, that is, black stains (using scalpels when possible, otherwise sterile swabs; ~50 mg material) and equivalent unstained control areas at about 10 cm of each black stain (using sterile swabs). At each sampling date, metabarcoding was carried out using three to six samples per wall surface condition. *Pseudomonas* isolation was performed using five rock wall samples (two black stains and three unstained areas).

Samples of collembola and rock walls (black stains and controls) used for sequencing were immediately transferred into liquid nitrogen before storage at −80°C, whereas samples for microbial isolation or isotopic analysis were kept à 4°C.

### Extraction of DNA, collembola identification and Illumina sequencing

Pools of entire collembola specimens (from 1 to 42 individuals per pool = per black stain) were crushed using 1‐mm diameter beads in ATL lysis buffer (Qiagen, Hilden, Germany) containing 20 mg.ml^−1^ lysozyme (Euromedex, Strasbourg, France) and homogenized for 10 s in a Mini‐beadbeaterTM (BioSpec Products, Bartlesville, OK). After 2 h at 37°C, 20 μl of proteinase K (Qiagen, 20 mg.ml^−1^) was added and samples incubated 4 h under agitation (300 rpm) at 56°C to lyse arthropod tissues completely. DNA was extracted with Qiagen DNeasy Blood and Tissue kit (Qiagen) following the manufacturer's recommendations for both Gram‐negative and Gram‐positive bacteria (elution with 12 μl).

Extraction of DNA from cave wall samples was done as described (Alonso et al., 2018), using the FastDNA SPIN Kit for Soil (MP Biomedicals, Illkirch, France), following the manufacturer's instructions. Elution was performed using two 50‐μl volumes, and final DNA concentration was measured using the Qubit dsDNA BR Assay Kit (Thermo Fisher Scientific, Eugene, OR) according to manufacturer's instructions. DNA extracts were stored at −20°C.

For taxonomic identification of collembola (done on 11 samples), a 708‐bp fragment flanking the mitochondrial cytochrome c oxidase subunit 1 (*cox1*) gene was amplified using 50 ng of DNA matrix and primers LCO1490 33 (5′‐GGTCAACAAATCATAAAGATATTGG‐3′) and HCO2198 (5′‐TAAACTTCAGGGTGACCAAAAAATCA‐3′) (Folmer et al., 1994). Amplification was performed in 25‐μl volumes containing 1× Q5 buffer (New England Biolabs, Ipswich, MA), 0.2 μM of primers (Invitrogen), 40 μM of dNTP (Applied Biosystems, Waltham, MA), 0.2 mg.ml^−1^ of Bovine Serum Albumin (New England Biolabs) and 0.35 U of Q5 DNA polymerase (New England Biolabs). PCR was performed at 94°C for 5 min, followed by 40 cycles of 94°C for 40 s, 50°C for 40 s, 72°C for 50 s and 72°C for 10 min. Sanger sequencing was performed (Biofidal company, Lyon, France) and sequences blasted against NCBI nr‐database (Johnson et al., 2008).

For Illumina MiSeq sequencing of cave wall and collembola DNA extract, PCR was done using primers 341F (5′‐CCTACGGGNGGCWGCAG‐3′) and 805R (5′‐GACTACHVGGGTATCTAATCC‐3′) for the V3–V4 region of bacterial 16 S rRNA genes (Klindworth et al., 2013), and ITS3_KYO2 (5′‐GATGAAGAACGYAGYRAA‐3′) and ITS4 (5′‐TCCTCCGCTTATTGATATGC‐3′) for the fungal internal transcribed spacer ITS2 (Toju et al., 2012). In addition, cave wall DNA extracts were also assessed using primers 18 S_0067a_deg (5′‐AAGCCATGCATGYCTAAGTATMA‐3′) and NSR399 (5′‐TCTCAGGCTCCYTCTCCGG‐3′) for eukaryotic 18 S rRNA genes (Dollive et al., 2012). Sequencing was performed by Fasteris company (Geneva, Switzerland), using 10 ng DNA and Illumina MiSeq (2 × 300 bp, paired‐end chemistry), to reach ≥70,000 paired‐end reads per sample.

### Bioinformatic analysis of Illumina data

For each gene marker, the paired‐end reads were demultiplexed by removing adaptators and all primer‐complementing sequences with two mismatches (or more) with original primer sequences, using a proprietary Perl script from Fasteris company (Alonso et al., 2019). Sequence analyses were performed as described (Alonso et al., 2019). Briefly, clustering was done using Swarm (Mahé et al., 2014), based on a local clustering threshold level (instead of a global one) and an aggregation distance of 3 to identify operational taxonomic units (OTUs) (Alonso et al., 2019). The finer taxonomic level that was reached corresponded to the genus or the species depending on the taxa. Taxonomic affiliation of OTUs at phylum, class, genus and/or species level was carried out automatically in the FROGS pipeline (Escudié et al., 2017), using RDP Classifier (Lan et al., 2012; Wang et al., 2007) against (i) the 119 SILVA database (Pruesse et al., 2007) for bacteria, (ii) the 123 SILVA database for eukaryotes, and (iii) the UNITE database for fungal ITS2 (Kõljalg et al., 2013).

### *Isolation and characterization of* Pseudomonas

For *Pseudomonas* isolation, swabs were vortexed in 0.9% NaCl solution, followed by spread plating onto S1 selective medium (Gould et al., 1985) and incubation at 12°C. Colonies were then grown in King's B medium (King et al., 1954), and DNA extraction was carried out using NucleoSpin Tissue (Macherey‐Nagel, Düren, Germany), following the manufacturer's instructions with support protocol for bacteria. Bacterial 16 S ribosomal fragments were amplified using primers pA (5′‐AGAGTTTGATCCTGGCTCAG‐3′) and pH (5′‐AAGGAGGTGATCCAGCCGCA‐3′) (Edgar, 2004) in 50‐μl reaction volumes containing 10× PCR buffer, 50 mM MgCl_2_, 2 mM dNTP, 10 μM of each primer, 1 unit of Taq polymerase (Invitrogen, Cergy‐Pontoise, France) and 1 μl of DNA solution. PCR was performed at 94°C for 5 min, followed by 35 cycles of 94°C for 30 s, 62.4°C for 30 s, 72°C for 40 s and 72°C for 3 min. PCR products were sequenced using Sanger methodology (Biofidal company, Lyon, France) and sequences were blasted against NCBI nr‐database (Johnson et al., 2008) to confirm affiliation to *Pseudomonas*.

### *Black fungi inhibition by* Pseudomonas

The inhibitory effect of 8 *Pseudomonas* isolates on 10 Lascaux black fungi (Table S1) was assessed with a dual culture protocol on solid medium. Each bacterium was grown 24 h in King's B broth and 5 μl of culture streaked 2 cm away from the centre of CYM plates (per litre: 10 g maltose, 20 g glucose, 2 g yeast extract, 2 g tryptone, 0.5 g MgSO_4_, 4.6 g KH_2_PO_4_; Sun et al., 2001 with 15 g agar), making a 2‐cm‐long line. A 5‐mm CYM fungal plug was then placed at the centre of each plate. As controls, CYM plates were inoculated only with fungi. The plates (two per treatment) were placed at 12°C in the dark during 15–30 days and growth inhibition was measured.

### Carbon isotope analyses and assimilation/dissemination experiments

A stable isotope analysis was performed on black stains from the Apse in June 2015. Five black stains currently colonized by collembola, four black stains that had been colonized years ago but not in recent times and four black stains without documented colonization history were sampled (using sterile scalpels), along with collembola (using sterile insect mouth aspirators) present on the five colonized stains. Stain samples were acidified with 2 N HCl to remove inorganic carbon, rinsed with ultrapure water and oven dried (70°C during 48 h), and weighed in tin capsules. Collembola were dried at 50°C for at least 48 h and each was weighed in a tin capsule (dry mass from 10 to 200 μg.individual^−1^).

The assimilation of organic carbon by *Folsomia candida* line HA (provided by Thomas Tully, iEES UPMC, Paris, France) was quantified using ^13^C/^12^C ratio. As ^13^C labelling of black stains was not possible, collembola were ^13^C enriched by feeding on ^13^C‐labelled glucose during 10 days (at 12°C) and placed 3 days at 12°C on water agar (15 g.l^−1^) containing a black stain sample, a plug of black fungus, or 5 μl of *Pseudomonas* culture (isolated from the Apse) in King's B medium. The black stain samples (approximately 2 cm^2^) were taken with a scalpel from the banks of the Passage (sample La852), the bottom of the wall of the Nave (La854) or the end of the Apse (Absidiole; La853). The black fungi were Lascaux isolates identified (ITS2 sequencing) as *Exophiala angulospora* (from a black stain in the second compartment of Airlock‐1 entrance zone), *Exophiala castellanii* (from a purple stain in the Apse), *Ochroconis lascauxensis* (from a black stain in the second compartment of Airlock‐1 entrance zone), and *Minimelanolocus* sp. (from a black stain in the Nave). For *Pseudomonas*, once the 5 μl of liquid King's B cultures had been placed on the agar, the bacteria were left 24 h to grow, which generated well‐developed bacterial colonies (diameter close to 5 mm; i.e. about the size of the food pellets that collembola were fed with during rearing) before collembola were added.

Two Petri dishes were used per treatment. The collembola from a given plate were transferred onto a new plate containing water agar and the plates incubated 3 days in the dark at 12°C. Whole collembola were harvested and used for stable isotope analysis, and both series of plates were assessed for microbial growth after 2 days at 12°C.

Stable carbon isotope ratios (^13^C/^12^C) were measured by continuous flow stable isotope ratio mass spectrometer (CF‐IRMS) using a Isoprime 100 (Elementar UK, Manchester, UK) mass spectrometer interfaced with a Vario PyroCube elemental analyser (Elementar Analysensysteme, Hanau, Germany). ^13^C/^12^C ratios were expressed as δ in parts per thousand (‰) and referenced to Vienna Pee Dee Belemnite (VPDB) standard. Data were calibrated against IAEA‐CH3 and IAEA‐CH6 international reference materials. The analytical precision achieved for aspartic acid and casein in‐house standards analysed along with the samples was better than 0.1 ‰ (±standard deviation).

For analysis of dissemination potential, four fungal colonies were randomly chosen per plate (i.e. per treatment, eight colonies from the first series of plates and eight others from the second). Each was transferred (as agar plug) on Dichloran Rose Bengal Chloramphenicol selective medium for 1 month at 12°C. For strains that did not grow on this medium, the agar plug was transferred onto CYM agar for 1 month at 12°C. All fungi were then grown on CYM medium at 22°C for 15 days to produce sufficient biomass. DNA extraction, PCR amplification of fungal ITS2 and Sanger sequencing were performed, as described above.

### Statistical analyses

To compare cave wall samples, a normalization procedure was applied for randomly resampling down to 6000, 26,674 and 21,074 Illumina sequences per sample in the bacteria, micro‐eukaryotes and fungi datasets, respectively. Sampling efficacy was assessed by computing rarefaction curves (Figure S2). Alpha diversity at OTU level was assessed using Chao 1 index (Chao, 1987), Shannon's H′ index (Shannon, 1948) and Simpson 1‐D index (Simpson, 1949), computed with Paleontological Statistics (PAST) software v3.14 (Hammer et al., 2001). Microbial community structure was investigated based on the Bray–Curtis similarity index (Bray & Curtis, 1957) after square‐root transformation of data (to avoid over‐dominance effects), using VEGAN package (http://cran.r-project.org/web/packages/vegan/index.html) in R. First, microbial communities were compared by non‐metric multidimensional scaling (NMDS), using PAST v3.14. Second, permutation multivariate analysis of variance using distance matrices (PERMANOVA) was carried out using adonis in VEGAN package to identify differences (*p* < 0.05) in overall community composition in phyla or classes and to confirm NMDS findings.

Analysis of variance (ANOVA) and Tukey HSD tests were performed to compare the number of OTUs or microbial diversity indices inside versus outside black strains (*p* < 0.05). Pearson's Chi‐squared tests in R were used for the proportions of phyla and genera in different microbial communities (*p* < 0.05). *t*‐tests (with Bonferroni correction for multiple comparisons) were used for stable isotope ratios (*p* < 0.05).

## RESULTS

### Specific fungal and micro‐eukaryotic diversities in black stains

**FIGURE 1:**
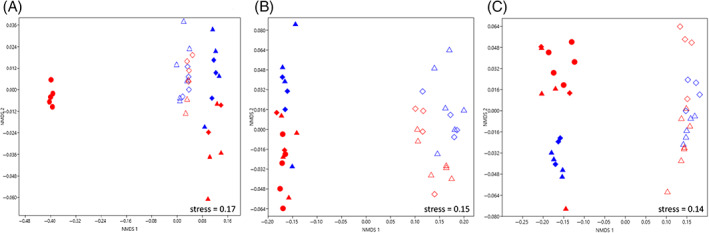
NMDS comparison of microbial communities present in black stains and nearby unstained parts of Lascaux's Apse. Samples from black stains (full symbols) and nearby unstained parts (empty symbols) were taken from the left (red) and right (blue) walls of Lascaux's Apse in June 2015 (circles), June 2016 (triangles) and December 2016 (diamonds), based on the relative proportion of both phyla and classes. (A) Fungal community (ITS2 regions). Differences were found when comparing (i) black stains on the left wall in June 2015 to the same stains at other sampling dates (PERMANOVA *F*_2,27_ = 14.5, *p* = 0.001, *R* ^2^ = 0.31), or (ii) these black stains on the left wall in June 2015 to black stains on the right wall at any of the samplings carried out (June 2016 or December 2016). In contrast, unstained parts next to black stains did not differ between left and right walls. (B) micro‐eukaryotic community (18S rRNA genes). Black stains (but not neighbouring unstained parts) differed between left and right walls (PERMANOVA *F*_1,30_ = 3.8, *p* = 0.01, *R* ^2^ = 0.04), and black stains (but not neighbouring unstained parts) from different sampling dates could differ (PERMANOVA *F*_2,30_ = 7.6, *p* = 0.001, *R* ^2^ = 0.16). (C) Bacterial community (16S rRNA genes). The bacterial community structure differed when comparing left walls to right walls (PERMANOVA *F*_1,29_ = 3.73, *p* = 0.018, *R* ^2^ = 0.04) for black stains (but not for unstained parts), whereas the effect of the sampling date was not significant.

ITS metabarcoding was used to test the hypothesis that a specific fungal community could establish in black stains in Lascaux's Apse, and indeed stains displayed a very distinct fungal community in comparison with unstained parts nearby, based on NMDS (PERMANOVA *F*_1,27_ = 28.4, *p* = 0.001, *R* ^2^ = 0.31; Figure 1A). At the larger scale of the whole micro‐eukaryotic community (18S rRNA gene analysis), black stains also differed from neighbouring unstained parts (PERMANOVA *F*_1,30_ = 36.8, *p* = 0.001, *R* ^2^ = 0.40; Figure 1B).

### Fungi with pigmentation potential are well established in black stains

**FIGURE 2:**
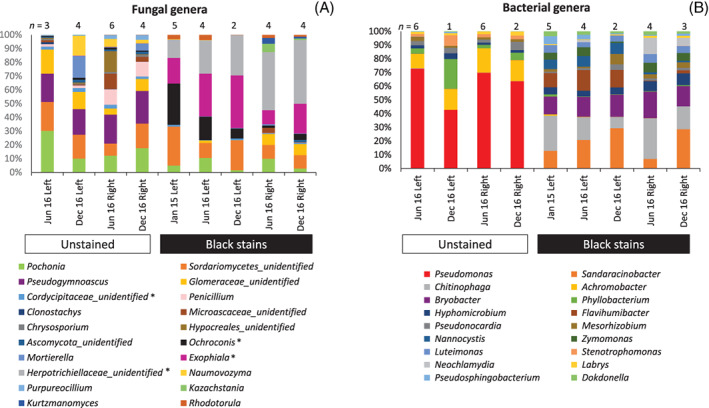
Community composition at genus level for fungi and bacteria in black stains and nearby unstained parts of Lascaux's Apse. Samples from the left (‘Left’) and right (‘Right’) walls were taken in June 2015 (‘Jun 15’), June 2016 (‘Jun 16’) and December 2016 (‘Dec 16’). Unstained parts did not yield sufficient DNA for analysis in June 2015. For visual clarity, only genera representing more than 1% of sequences are indicated, but a complete version including all genera is available in Figure S4. Each histogram is the average from one to six samples (indicated in each case). (A) Fungal community (ITS2 regions). Fungi with the potential to produce black pigments are indicated with an asterisk. In unstained parts of the Apse sampled in the vicinity of black stains, the genus taxonomic profile of fungi differed when comparing left versus right wall samples (Chi‐squared test, *p* < 0.0001), but comparatively, it was less variable in time across the 1.5 years of the study. (B) Bacterial community (16S rRNA genes)

Only a subset of fungi is documented to produce black pigments (Alonso et al., 2018; Saiz‐Jimenez, 2012), and therefore we monitored more specifically the taxonomic composition of the fungal community (ITS genes) to consider taxa with pigmentation potential. We show that the genus profile of fungi differed strongly in black stains versus neighbouring unstained parts (Chi‐squared test, *p* = 0.0001), for example, with fewer *Pseudogymnoascus* in black stains, regardless of the Apse wall investigated (Figures 2A and S3A). However, the fungal profile differed also when comparing black stain samples that had been taken (i) at different sampling events or (ii) from different walls. These findings were also made when considering only the subsets of (i) pigmented fungal taxa (e.g. *Ochroconis* represented 7.1%–29.6% of fungal sequences in left‐wall black stains, 1.7%–4.1% in right‐wall stains, versus only 0.7%–1.4% in left‐ and right‐wall unstained parts) and (ii) fungal taxa containing both pigmented and non‐pigmented strains (*Exophiala* and Herpotrichiellaceae represented a total of 31.6%–67.5% of fungal sequences in black stains versus only 0.07%–0.87% in unstained parts) instead of the whole fungal community (Figure S4). In contrast to *Ochroconis lascauxensis*, the pigmented fungus *O. anomala* was evidenced at very low numbers of sequences, in accordance with previous data (Martin‐Sanchez et al., 2013; Martin‐Sanchez, Nováková, et al., 2012b), whereas the *Fusarium* genus implicated in earlier outbreak (white mycelial growth, early 2001) (Bastian et al., 2010) was found in <0.15% of sequences. The genus taxonomic profile of all micro‐eukaryotes (18S rRNA genes) pointed to the same results regarding the prevalence of fungi with pigmentation potential in black stains (Figure S5).

**FIGURE 3:**
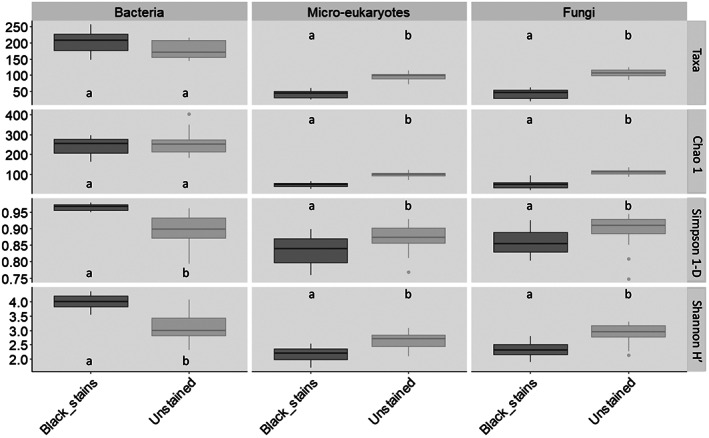
Number of taxa (Taxa), Chao1 index of OTU richness (Chao1), Simpson's index of diversity (Simpson 1‐D) and Shannon index of OTU diversity (Shannon H′) for bacteria, micro‐eukaryotes and fungi in black stains and nearby unstained parts sampled in Lascaux's Apse walls. Data were combined for the left and right walls sampled in June 2015, June 2016 and December 2016. Significant differences between conditions are shown with lowercase letters (based on ANOVA and Tukey's tests *p* < 0.05).

The differences in black fungus prevalence might be accounted for if black stains had contained the same amounts of pigmented fungi but lower amounts of total fungi than unstained parts, yet qPCR data of 18S rRNA genes showed that black stains (10,291 ± 30 copies of 18S rRNA genes) were equally populated than unstained parts (9702 copies ± 73 copies of 18S rRNA genes) (Figure S6). It means that the higher abundance of pigmented fungal taxa in black stains was not a bias due to lower amounts of total microorganisms, but rather that it had resulted from black fungi proliferation. This higher abundance of black fungi resulted in lower α‐diversity indices of the fungal community in black stains than in unstained parts (ITS genes; Figure 3). In contrast, fungal α‐diversity indices did not differ significantly when comparing sampling times or right and left walls (Figure S7).

In summary, black stains exhibited a very specific fungal microbiome, which was characterized by the prevalence of *Ochroconis* and other taxa of pigmented fungi, whereas these fungi were in a tiny minority on healthy surfaces. This provides further evidence that the development of black pigmented fungi is associated with black stain formation in the Apse of Lascaux.

### Bacterial diversity differs in black stains versus unstained surfaces

Since fungal dynamics may be influenced by interactions with bacteria (Frases et al., 2006; Frey‐Klett et al., 2011), we also focused on bacterial diversity, using 16S metabarcoding. The bacterial community structure differed when comparing black stains with neighbouring unstained parts (PERMANOVA *F*_1,29_ = 53.9, *p* = 0.001, *R* ^2^ = 0.53) (Figure 1C).

In unstained parts of the Apse, the bacterial community was dominated by the proteobacterial genus *Pseudomonas*, which represented a very large proportion of the sequences (i.e. 34%–63%), regardless of the wall or sampling date (Figures 2B and S3B). *Pseudomonas* aside, the rest of the genus taxonomic profile of bacteria was rather comparable in all unstained part samples (Figure S8). In contrast, the relative abundance of *Pseudomonas* was much lower in black stains, as they accounted for only 0.01%–0.11% of all bacterial sequences, indicating strong negative selection.

When *Pseudomonas* data were removed, so as to focus on the rest of the bacterial community, the genus taxonomic profile of bacteria differed between black stains and unstained surfaces (Figure S8). In particular, four bacterial genera were more abundant in unstained parts than in black stains, that is, *Achromobacter* (18.5%–41.1% vs. 0.02%–0.60%), *Phyllobacterium* (5.8%–26.5% vs 0.2–1.1%), *Pseudonocardia* (4.8–11.7% vs 0.67%–2.38%), *Stenotrophomonas* (4.9%–9.3% vs. 0%–0.01%), and three other bacterial genera were more abundant on black stains than on unstained parts, that is, *Sandaracinobacter* (3.60%–17.0% vs. 0–0.17%), *Chitinophaga* (4.8%–16.5% vs. 0.19%–0.49%) and *Bryobacter* (7.82%–10.4% vs. 0.04%–0.24%).

As for fungi, these differences in the abundance of particular genera were not due to lower amounts of total bacteria in black stains, as indicated by qPCR data of 16 S rRNA genes (Figure S6). Black stains displayed higher Simpson and Shannon indices in comparison with unstained parts (Figure 3), meaning that the much lower abundance of *Pseudomonas* in black stains coincided with proliferation of many other bacterial taxa (as shown in Figure S8).

### Pseudomonas *strains outside stains can inhibit pigmented fungi*

Since the high prevalence of pseudomonads outside black stains coincided with a low abundance of black fungi (Pearson correlation coefficient of −0.98 between sequence percentages of *Ochroconis lascauxensis* and *Pseudomonas* spp., *p* = 0.025), it raised the possibility that the pseudomonads could limit the establishment of these fungi in unstained parts of the Apse walls. Other bacteria might also be implicated, but we focused on *Pseudomonas* because of its outstanding prevalence outside stains. *Pseudomonas* isolates were readily obtained from unstained wall samples, but only one could be found from black stains, as expected considering their very low abundance on stains.

Dual‐confrontation experiments were carried out on agar plates with eight black fungi from Lascaux and seven *Pseudomonas* isolates from unstained parts of the Apse. This indicated that one *Pseudomonas* inhibited a single black fungus only (i.e. *Ochroconis lascauxensis*), but the six others inhibited three to five black fungi each (among *Acremonium nepalense*, *Alternaria alternata*, *Doratomyces* sp., *Exophiala angulospora*, *Ochroconis lascauxensis* and/or *Minimelanolocus* sp.) (Figure S9 and Table S1). The black stain isolate inhibited only *Doratomyces* sp., thus its inhibition potential was low.

### *Black fungi rather than* Pseudomonas *are consumed by cave collembola*

**FIGURE 4:**
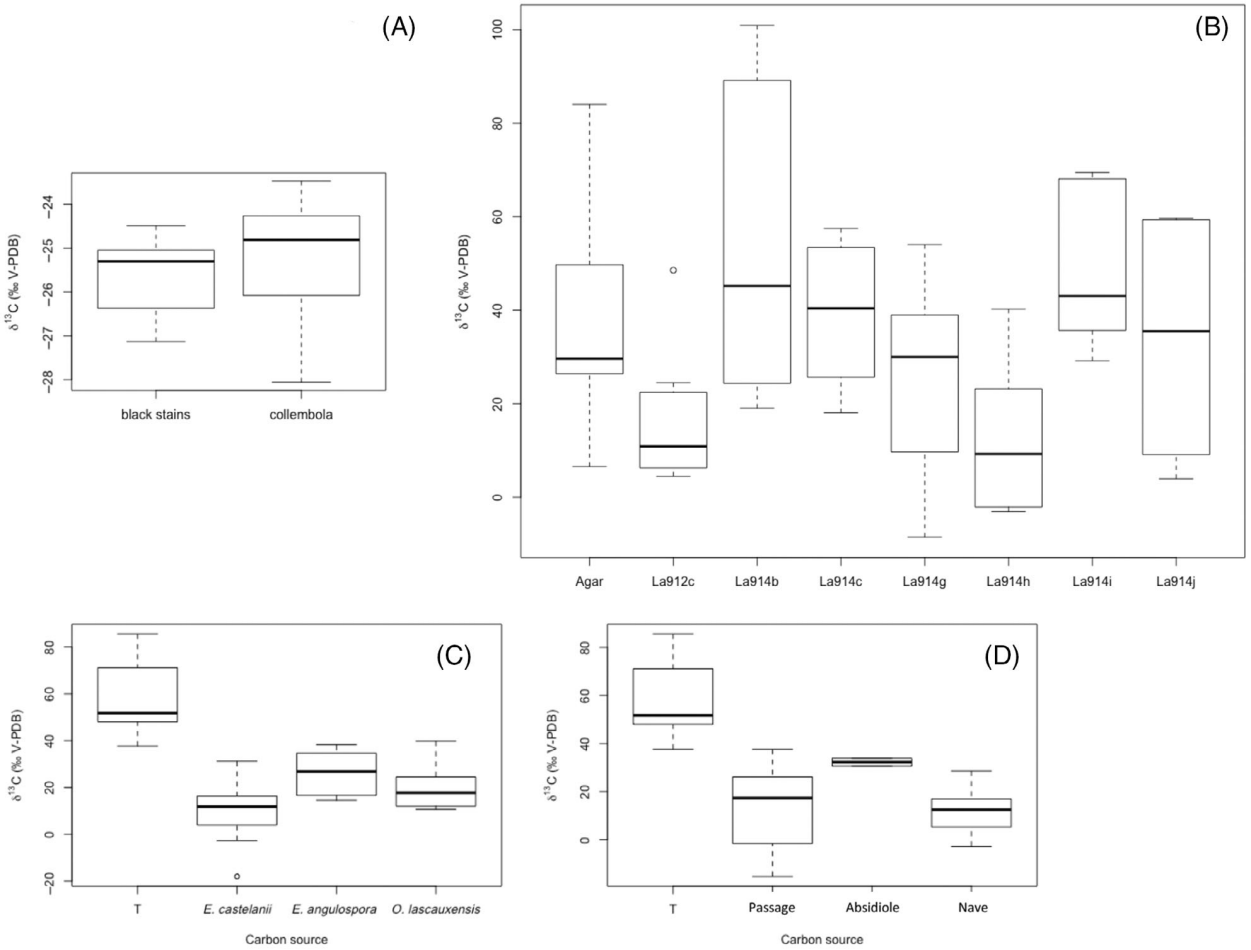
Carbon isotopic analysis of microbial assimilation by the collembola *Folsomia candida*. (A) Natural δ^13^C values of 13 black stains from Lascaux's Apse and 11 *F. candida* samples (of at least five collembola individuals each) collected on black stains in the cave; the difference in δ^13^C values of black stains and *F. candida* was not significant. (B) δ^13^C values of collembola *F. candida* line HA following ^13^C enrichment and incubation without carbon source (i.e. ‘Agar’ control) or in the presence of Lascaux pseudomonads La912c isolated from black stain and La914b, La914c, La914g, La914h, La914i and La914j isolated from unstained parts of the Apse; the δ^13^C value of ^13^C‐enriched collembola was significantly reduced in presence of strains La912c or La914h but not with the five other strains. (C) δ^13^C values of collembola *F. candida* line HA following ^13^C enrichment and incubation without carbon source (i.e. ‘T’ control) or in the presence of Lascaux black fungi *Exophiala castellanii*, *E. angulospora* and *O. lascauxensis*; the δ^13^C value of ^13^C‐enriched collembola was significantly reduced with the three fungi. (D) δ^13^C values of collembola *F. candida* line HA following ^13^C enrichment and incubation without carbon source (i.e. ‘T’ control) or in presence of black stain samples from the banks of the passage, the far end of the Apse (termed Absidiole) and the bottom of the wall of the Nave; the δ^13^C value of ^13^C‐enriched collembola was significantly reduced with black stains from Passage and Nave, but the trend was not significant at *p* < 0.05 with black stain from the Absidiole.

Collembola on black stains (Figure S1) were identified as *F. candida* by *cox1* sequencing of 11 collembola samples. Earlier experiments carried out in vitro showed that *F. candida* was able to graze on black fungi and probably to feed on them (Bastian et al., 2010), but these findings relied on laboratory experiments and evidence of assimilation of fungal constituents in the collembola biomass was lacking. To assess whether collembola could assimilate organic C from black fungi (rather than from pseudomonads), we performed isotopic assays. First, we compared the δ^13^C values measured directly in black stains of the Apse (δ^13^C ranging from −28.0 to −23.8‰) and in *F. candida* collected from the same black stains (δ^13^C ranging from −28.1 to −23.5‰), which showed that the carbon isotope signatures of stains and collembola were similar (Welch *t*‐test, *p* = 0.2191; Figure 4A), strengthening the hypothesis (Bastian et al., 2010) that *F. candida* thriving on black stains was actually feeding on these fungi.

Second, direct evidence for effective feeding of *F. candida* on black fungi was sought by monitoring ^12^C assimilation, following an artificial ^13^C enrichment of the animals during rearing. Subsequent in vitro feeding experiment of ^13^C labelled collembola on non‐labelled, laboratory‐grown black fungi *Exophiala castelanii* (δ^13^C = −24.6‰ ±1.2‰), *Exophiala angulospora* (δ^13^C = −23.1‰ ± 0.5‰) and *Ochroconis lascauxensis* (δ^13^C = −21.9‰ ± 0.1‰) resulted into a significant decrease (Welch *t*‐tests with Bonferroni correction, all *p* < 0.001) in the δ^13^C values of *F. candida* from 54.2‰ ± 15.8‰ to (i) 9.4‰ ± 15.7‰ when using *Exophiala castelanii*, (ii) 25.9‰ ± 19.5‰ when using *Exophiala angulospora*, and (iii) 19.8‰ ± 9.9‰ when using *Ochroconis lascauxensis* (Figure 4C). This demonstrates that collembola had assimilated non‐labelled carbon from these fungi. Gut darkening was also observed visually during the experiment (Figure S1C), as expected from Bastian et al. (2010).

Third, to consider actual cave conditions more directly, the experiment was repeated with black stain samples (whose δ^13^C was −25.5‰ ± 0.5‰) taken directly from different rooms of Lascaux (including the Apse). Results showed a significant decrease in the δ^13^C values of ^13^C‐enriched collembola (and gut darkening took place) for stains originating from the Passage or the Nave (*p* < 0.0001 each), whereas this trend was not statistically significant when black stain from the Apse was used (Figure 4D).

Fourth, since fewer pseudomonads were found on black stains compared with unstained parts, an exploratory assessment of these pseudomonads as potential carbon source of *F. candida* was also done. The δ^13^C value of ^13^C‐enriched collembola (37.8‰ ± 23.3‰; whole bodies studied) was reduced to only 16.5‰ ± 14.8‰ after exposure 3 d to the black stain pseudomonad La912c (itself at δ^13^C = −23.7‰ ± 0.1‰) or to the pseudomonad La914h from unstained surface (12.2‰ ± 15.5‰), but values of similar magnitude as in collembola were found with any of the five other *Pseudomonas* isolates originating from unstained walls (Figure 4B). This means that collembola did not feed significantly on most antagonistic pseudomonads from unstained parts.

### Collembola readily disseminate Lascaux microorganisms

Collembola are thought to promote fungal spread in caves (Martin‐Sanchez, Sanchez‐Cortes, et al., 2012), as shown with individual fungi (Bastian et al., 2010), but experimental demonstration of microbial dissemination potential from cave's black stains was lacking. Therefore, we tested their dissemination potential by feeding *F. candida* on laboratory‐grown black fungi or on cave's black stain samples placed on plates (Stage 1) and subsequently transferring them on fresh plates (Stage 2), so as to monitor which fungi had been disseminated. First, analysis of plates from both stages of the experiment showed that three of the four test fungi could be disseminated, on Stage 1 plates (for *E. castellanii*) or both Stage 1 and Stage 2 plates (for *Ochroconis lascauxensis* and *Exophiala angulospora*). A fourth test fungus, *Minimelanolocus* sp., could only be disseminated on Stage 1 plate. Additional culturable microorganisms presumably associated with collembola surfaces or guts were also disseminated, that is, bacteria (not characterized taxonomically) and fungi (belonging to genera *Alternaria*, *Cladosporium*, *Engyodontium* or *Sistotrema*). Second, the experiment carried out using black stain samples also showed dissemination of bacteria (not characterized taxonomically) and fungi on plates, including the fungi *Alternaria chlamydosporigena*, *Exophiala* sp. and *E. castellanii*, *Ilyonectria* sp., *Mortierella* sp., *Ochroconis* sp. and *Pyrenochaeta acicola* on Stage 1 plates, *Pochonia* sp. on Stage 2 plates, and *Minimelanolocus* sp. on both. Six of these taxa (*Ochroconis*, *Exophiala*, *E. castellanii*, *Mortierella*, *Minimelanolocus* sp.*, Pochonia*) had been identified in MiSeq data from black stains of the Apse. Thus, experimental evidence showed that *F. candida* has the capacity to disseminate Lascaux's black fungi.

Since collembola were identified as potential disseminators of microorganisms present in black stains, MiSeq sequencing was implemented to document the range of microorganisms associated with *F. candida* residing on black stains and assess whether microorganisms from black stains could be also evidenced in collembola. A total of 470 bacterial OTUs associated with collembola were identified, including the endosymbiont *Wolbachia* and *Pseudomonas*. As many as 369 of 382 bacterial OTUs found in black stains (i.e. 97%) were also evidenced in collembola sampled on these black stains (Figure S10). In addition, 148 fungal OTUs were documented, including *Acremonium nepalense*, *Exophiala castellanii*, *Ochroconis lascauxensis* (all with black pigmentation potential), and related *Alternaria eichhorniae* and *A. metachromatica* (whose pigmentation potential is not documented). Several types of fungi transported by collembola in the dissemination experiment were also evidenced in ITS2 MiSeq data for Apse collembola, that is, the two test fungi *Exophiala castellanii* and *Ochroconis lascauxensis*, as well as the genera *Alternaria*, *Engyodontium*, *Exophiala*, *Ilyonectria*, *Ochroconis* and *Pochonia* directly associated with collembola and/or observed on plates when black stain samples were studied for dissemination. More generally, 21 of 24 fungal OTUs found in black stains (i.e. 88%) were also evidenced in collembola sampled on these black stains (Figure S10). Thus, we established that many microbial taxa thriving in black stains, such as black pigmented fungi, were also associated with the *F. candida* collembola present on the stains.

## DISCUSSION

Black stains represent a major threat for conservation of Palaeolithic art in caves (Bontemps et al., 2021). Despite the hypotheses put forward on the contribution of pigmented fungi (Bastian et al., 2009, 2010) capable of melanin synthesis (De la Rosa et al., 2017; Martin‐Sanchez, Sanchez‐Cortes, et al., 2012), the formation and dynamics of these black stains are not well understood. Against this background, the current work established that the fungal community in Lascaux's Apse differed strongly between black stains and neighbouring unstained parts. Such differences had not been found in another Lascaux room termed the Passage (Alonso et al., 2018), but the black stains studied there had formed years before and are no longer active. In addition, variability of microbiota features across Apse walls and sampling times was of moderate magnitude, thereby facilitating the demonstration of significant differences between surface conditions. Microbial diversity of Lascaux's black stains had also been investigated prior to the development of new‐generation sequencing technology, but it was done using a very limited number of samples and without direct comparison with unstained controls (Bastian et al., 2009; Martin‐Sanchez, Nováková, et al., 2012a). In addition, we found that fungi with pigmentation potential, for example, *Ochroconis* (Alonso et al., 2018), were quite prevalent in the Apse's black stains but rare on unstained surfaces. This provides further evidence that the development of black pigmented fungi is associated with black stain formation in the Apse of Lascaux.

Microbiome specificity of black stains was not limited to the case of pigmented fungi and concerned also bacteria, as, for instance, the *Pseudomonas* genus highly dominant on healthy surfaces had almost disappeared in black stains. The *Pseudomonas* bacteria displayed inhibitory potential towards black fungi, suggesting that they (as well perhaps as other bacteria) might explain why black stains do not develop on a larger scale within Lascaux's Apse.

Cave anthropization favours collembola invasion (Alonso et al., 2019), and collembola (most presumably *Folsomia candida*) occurring on Lascaux's black stains have been reported for years (Bastian et al., 2010). 18S rRNA sequences pointing to *Folsomia* had been obtained by cloning‐sequencing from a black stain in Lascaux's Painted Gallery (Bastian et al., 2009), which was confirmed here by *cox1* sequencing. The use of isotopic analyses demonstrated that collembola could assimilate carbon from Lascaux's black fungi. *F. candida* from other ecosystems prefers black fungi (Böllmann et al., 2010; Scheu & Simmerling, 2004) over others, whereas they can even be repelled by certain fungi (Böllmann et al., 2010). Furthermore, isotopic analyses showed that these collembola could feed on Lascaux black stains. *F. candida* can also feed on bacteria, and bacteria could be seen in the gut and faecal pellets of laboratory‐reared specimens by scanning microscopy (Thimm et al., 1998). In the case of Lascaux, however, our experimental data showed that *F. candida* collembola did not assimilate organic carbon significantly from most antagonistic *Pseudomonas* strains tested.

Previous work had proposed that collembola have the potential to disseminate fungi via their faeces (Bastian et al., 2010; Sabatini et al., 2004). In addition, many dominant fungal phylotypes found in cave stains corresponded to taxa typically associated with arthropods (Bastian et al., 2009), suggesting that the latter could have contributed to fungal transmission. Finally, *F. candida* retrieved from cave displayed fungal conidia in the gut based on histological imaging (Smrž et al., 2015), whereas the presence of various bacteria associated with *F. candida* from soil have been documented by 16S fingerprinting (Czarnetzki & Tebbe, 2004). Here, this dissemination potential was confirmed based on experimental evidence obtained with individual black fungi from Lascaux and especially black stains themselves, indicating that *F. candida* can disseminate a range of cave's black fungi and promote founder effects.

MiSeq sequencing showed that a wide range of microbial taxa present in black stains (including black pigmented fungi such as *Ochroconis*) were also found associated with collembola collected from the same stains. Many of these microbial taxa were probably present on animal surfaces such as legs and buccal parts (as pointed by Dromph, 2001; Martin‐Sanchez, Sanchez‐Cortes, et al., 2012), based on (i) the recovery of taxa usually not associated with arthropod guts, for example, *Sandaracinobacter, Bryobacter*, *Dokdonella* and *Davidiella*, and (ii) the high number of microbial OTUs common to collembola samples and to black stains on which the collembola were present. Collembola might thus represent a microbial reservoir, raising the possibility that they could assist the establishment of pioneer microbial colonies as they wander on cave walls (Martin‐Sanchez, Sanchez‐Cortes, et al., 2012), contaminating unstained surfaces with their legs and depositing faecal pellets containing microorganisms, since geographic dissemination by faecal pellets can be extensive in vitro (Bastian et al., 2010). Accordingly, certain microorganisms may release volatile chemoattractants that facilitate their subsequent spread by *F. candida* (Becher et al., 2020).

Overall, this study points to the following scenario, which completes the propositions and data of Bastian et al. (2010) and Martin‐Sanchez, Sanchez‐Cortes, et al. (2012). The proliferation of *F. candida* collembola is favoured by grazing on pigmented fungi and black stain compounds, while the collembola have the potential to promote (i) the dissemination and development of black pigmented fungi, and (ii) more generally the establishment of a stain‐specific fungal community. Thus, it can be speculated that the new black stains that did form in recent years might have implicated occasional founder effects hypothetically resulting from collembola‐mediated microbial dissemination, perhaps at microsites where the pseudomonads (or other bacteria) present were less effective at inhibiting fungi. We also speculate that the elimination of pseudomonads taking place once black stains is established might result from maladaptation to the new abiotic conditions prevailing in the stain, as (i) collembola did not feed significantly in vitro on most (five of six) pseudomonads from unstained parts and (ii) black fungi did not interfere with growth of these pseudomonads when tested in vitro.

## AUTHOR CONTRIBUTIONS

**Lise Alonso:** Formal analysis (equal); investigation (equal); writing – original draft (equal). **Thomas Pommier:** Conceptualization (equal); investigation (equal); writing – review and editing (equal). **Laurent Simon:** Formal analysis (equal); investigation (equal). **Flavien Maucourt:** Investigation (equal). **Jeanne Doré:** Investigation (equal). **Audrey Dubost:** Data curation (equal); formal analysis (equal). **Van Trân Van:** Investigation (equal). **Guillaume Minard:** Formal analysis (equal); investigation (equal); writing – review and editing (equal). **Claire Valiente Moro:** Formal analysis (equal); writing – review and editing (equal). **Christophe J. Douady:** Conceptualization (equal); formal analysis (equal); writing – review and editing (equal). **Yvan Moënne‐Locco:** Conceptualization (equal); funding acquisition (equal); investigation (equal); project administration (equal); writing – review and editing (equal).

## CONFLICT OF INTEREST

The author declares that there is no conflict of interest.

## Supporting information


**TABLE S1.** Effect of *Pseudomonas* isolates from the Apse on growth of Lascaux black fungi (All Ascomycota). Isolate La912c was obtained from a black stain (indicated by grey background) and the seven others from untained parts of Apse walls. The ‘plus’ indicate fungus inhibition by *Pseudomonas* and ‘minus’ a lack of inhibition. NC, test not conclusive due to bacterial swarming in presence of certain fungi.

**Figure S1‐S10:** Supplementary Figures.

## Data Availability

Sequences are available from the European Nucleotide Archive (EMBL‐EBI, UK) with accession numbers PRJEB27522 (*cox1* genes from *F. candida*), PRJEB27258 (MiSeq sequences for wall samples), PRJEB27257 (MiSeq sequences for collembola samples) and PRJEB27281 (*Pseudomonas* strains).
