# Supplementary material for: Microbiome analysis in Lascaux Cave in relation to black stain alterations of rock surfaces and collembola
Source: Environ Microbiol Rep. 2022 Nov 24;15(2):80–91. doi: 10.1111/1758-2229.13133 (PMC10103860; doi:10.1111/1758-2229.13133)
Supplement: Supplementary file 1 — TABLE S1. Effect of Pseudomonas isolates from the Apse on growth of Lascaux black fungi (All Ascomycota). Isolate La912c was obtained from a black stain (indicated by grey background) and the seven others from untained parts of Apse walls. The ‘plus’ indicate fungus inhibition by Pseudomonas and ‘minus’ a lack of inhibition. NC, test not conclusive due to bacterial swarming in presence of certain fungi. [file EMI4-15-80-s001.docx]

| **TABLE S1** Effect of *Pseudomonas* isolates from the Apse on growth of Lascaux black fungi (All Ascomycota). Isolate La912c was obtained from a black stain (indicated by grey background) and the seven others from untained parts of Apse walls. The ‘plus’ indicate fungus inhibition by *Pseudomonas* and ‘minus’ a lack of inhibition. NC, test not conclusive due to bacterial swarming in presence of certain fungi. | | | | | | | | | |
| --- | --- | --- | --- | --- | --- | --- | --- | --- | --- |
| **Black fungi tested** | **Higher fungal taxonomy** | **La912c** | **La914b** | **La914c** | **La914e** | **La914g** | **La914h** | **La914i** | **La914j** |
| *Exophiala angulospora* | Class Chaetothyriomycetes, Order Chaetothyriales, Family Herpotrichiellaceae | - | - | - | - | - | + | - | - |
| *Exophiala castellanii* | Class Chaetothyriomycetes, Order: Chaetothyriales, Family Herpotrichiellaceae | - | NC | - | - | - | - | - | - |
| *Exophiala* sp. | Class Chaetothyriomycetes, Order: Chaetothyriales, Family Herpotrichiellaceae | - | - | - | - | - | - | - | - |
| *Ochroconis lascauxensis* | Class Dothideomycetes, Order Venturiales, Family Sympoventuriaceae | - | - | - | + | + | + | + | + |
| *Doratomyces* sp. | Class Sordariomycetes, Order Microascales, Family Microascaceae | + | + | + | + | + | - | NC | + |
| *Acremonium nepalense* | Class Sordariomycetes, Order Hypocreales, Family Hypocreaceae | - | + | + | + | + | + | - | - |
| *Minimelanolocus* sp. | Class Eurotiomycetes, Order Chaetothyriales, Family Herpotrichiellaceae | - | + | + | + | NC | + | - | + |
| *Alternaria alternata* | Class Dothideomycetes, Order Pleosporales, Family Pleosporaceae | - | + | - | + | - | - | NC | + |
| *E. angulospora* was isolated from a black stain in the second compartment of Airlock-1 entrance zone*, E. castellanii* from a purple stain in the Apse*, Exophiala* sp. from a black stain in the second compartment of Airlock-1 entrance zone, *O. lascauxensis* from a black stain in the second compartment of Airlock-1 entrance zone, *Doratomyces* sp. from unstained part on wall in Passage, *A. nepalense* from a black stain in the second compartment of Airlock-1 entrance zone, *Minimelanolocus* sp. from a black stain in the Nave, and *A. alternata* (LRMH isolate) from the air. | | | | | | | | | |
