# Supplementary material for: Microbiome analysis in Lascaux Cave in relation to black stain alterations of rock surfaces and collembola
Source: Environ Microbiol Rep. 2022 Nov 24;15(2):80–91. doi: 10.1111/1758-2229.13133 (PMC10103860; doi:10.1111/1758-2229.13133)
Supplement: Supplementary file 2 — Figure S1‐S10: Supplementary Figures. [file EMI4-15-80-s002.pdf]

Fig. S1 Illustrations of black stains and collembola. They include photographs of black stains in the Apse (**D, H, L**), Dino-Lite microscope photographs of black stains (**A, B, E, F, G**) and neighboring unstained parts (**I, J, K**) on right walls (**A, E, I**), left walls (**B, F, J**) and the Absidiole area (**G, K**) of the Apse, and binocular microscope photograph of *Folsomia candida* collembola on a black fungal colony on laboratory medium (**C**). Collembola can be seen on black stains (**A**, **B**, **F**). Macroscopically, many black stains are entirely black whereas others also contain areas of lighter color (visible also in microscope photograph **F**), yet most display some level of patchiness when examined by microscopy. During the feeding experiment *in vitro*, darkening of collembola gut can be seen thanks to the transparency of the animal tissues, once the black fungus was grazed (**C**). In **D**, collembola can be seen as small white spots on the black stain.

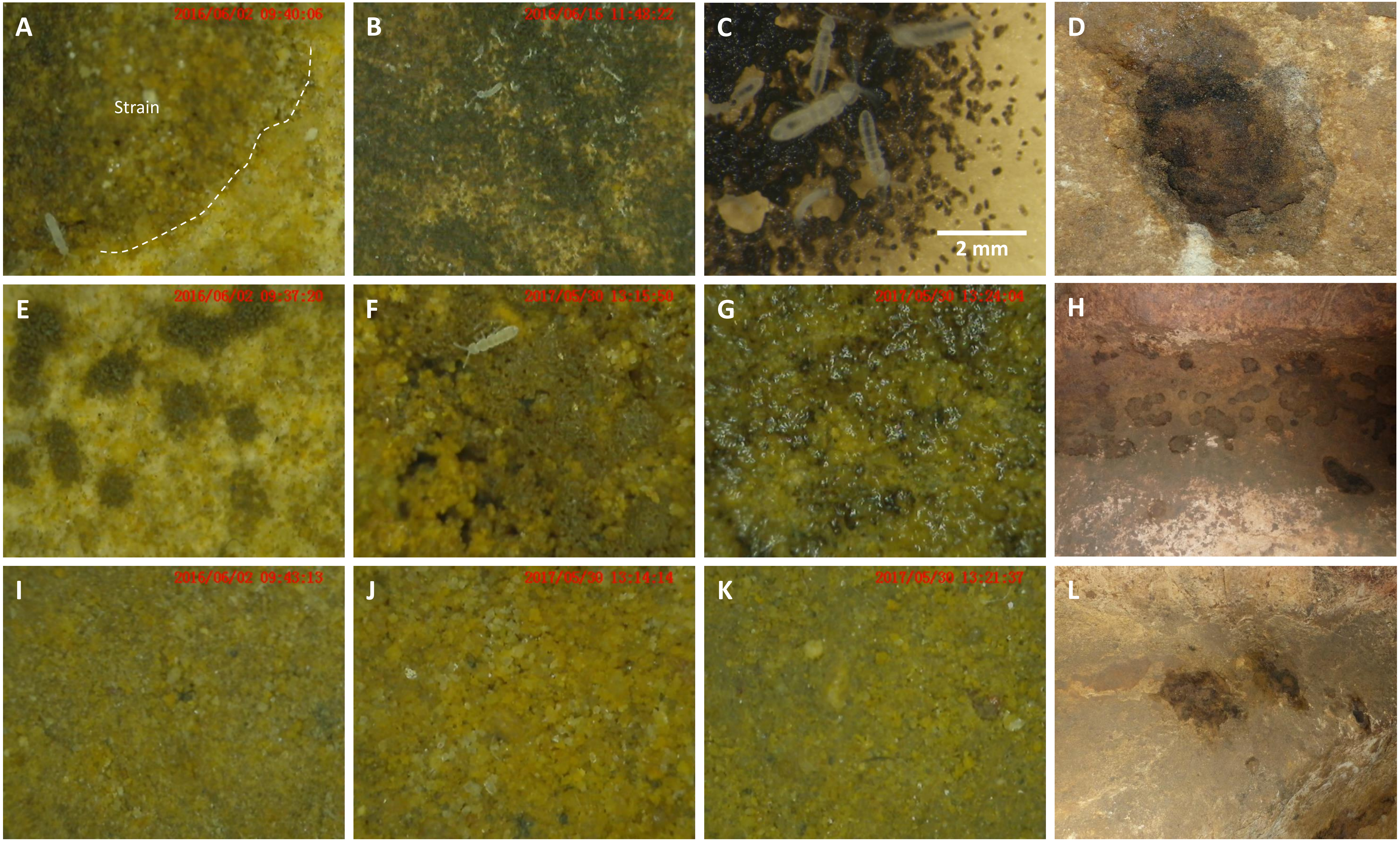
A
2016/06/02 09:40:06
Strain
B
2016/06/16 11:48:22
C
2 mm
D
E
2016/06/02 09:37:20
F
2017/05/30 13:15:50
G
H
I
2016/06/02 09:43:13
J
2017/05/30 13:14:14
K
2017/05/30 13:21:37
L

Fig. S1

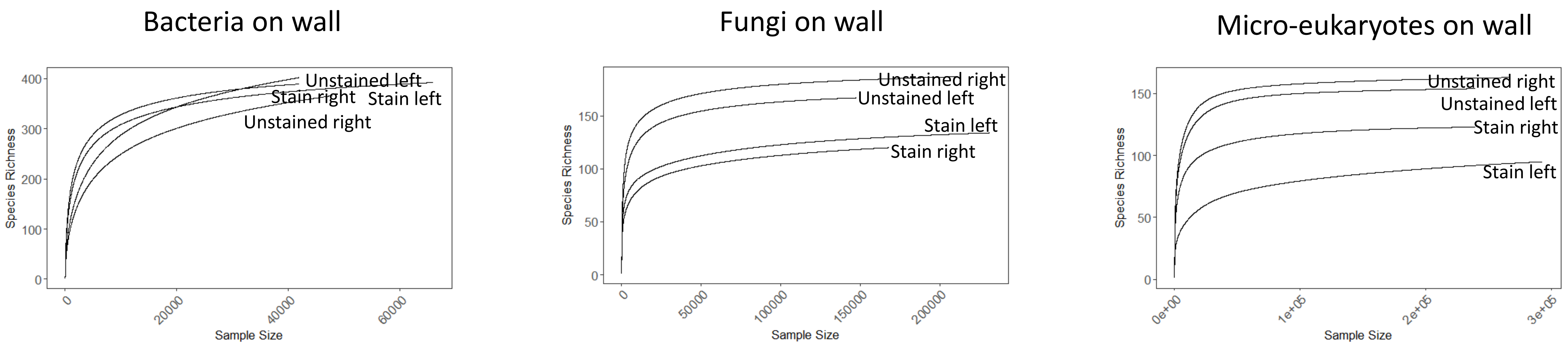

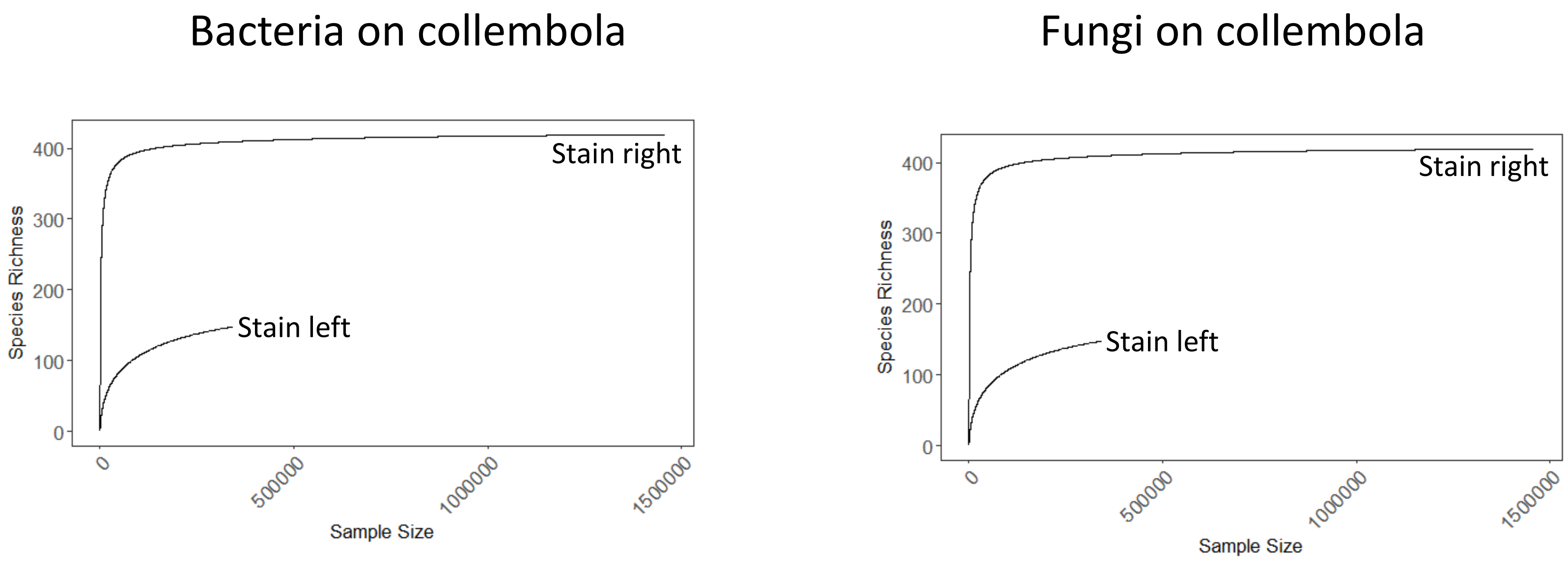

Fig. S2

Fig. S2 Rarefaction curves at OTU level for cave wall samples in the case of bacteria (16S rRNA gene dataset), micro-eukaryotes (18S rRNA gene dataset), fungi (ITS2 dataset) and for collembola samples in the case of bacteria (16S rRNA gene dataset) and fungi (ITS2 dataset), based on observed data. Wall samples originated from black stains and neighbouring unstained parts (from the left and right walls), whereas collembola samples originated from black stains (both left and right walls combined). Rarefaction curves reached an asymptote in 2 out of 4 cases (wall bacteria), all 4 cases (wall micro-eukaryotes), all 4 cases (wall fungi), 1 of 2 cases (collembola's bacteria) and 1 of 2 cases (collembola's fungi).

Fig. S3 Community composition at genus level for fungi and bacteria in black stains and nearby unstained parts of Lascaux's Apse (blown-up version of Fig. 2). Samples from the left ('Left') and right ('Right') walls were taken in June 2015 ('Jun 15'), June 2016 ('Jun 16') and December 2016 ('Dec 16'). All genera evidenced are shown. Each histogram is the average from 1-6 samples (indicated in each case). **A**, Fungal community (ITS2 regions). Fungi with the potential to produce black pigments are indicated with an asterisk. In unstained parts of the Apse sampled in the vicinity of black stains, the genus taxonomic profile of fungi differed when comparing left vs right wall samples (Chi-squared test, $P < 0.0001$), but comparatively it was less variable in time across the 1.5 years of the study. **B**, Bacterial community (16S rRNA genes).

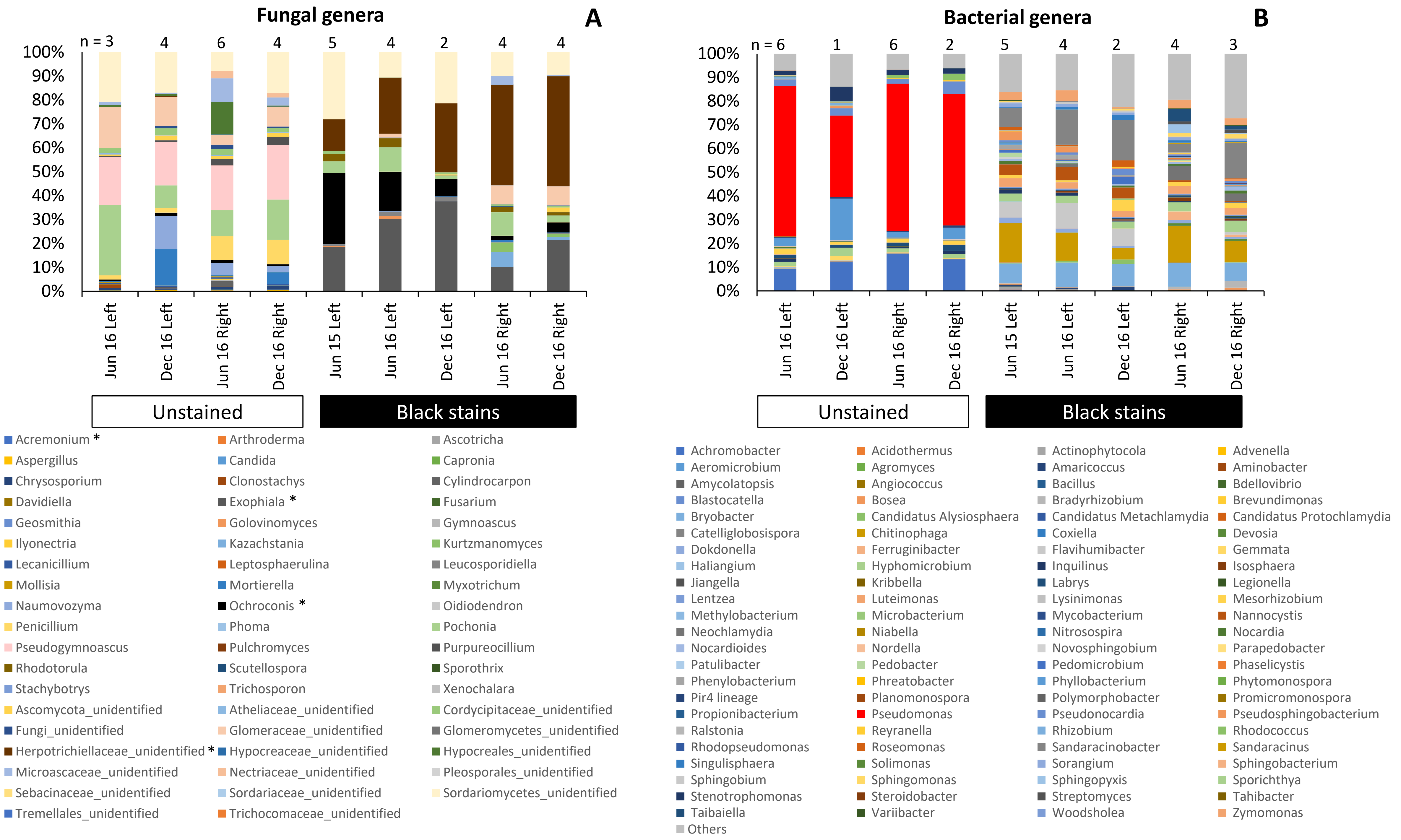
Fungal genera
A
n = 3
4
6
4
5
4
2
4
4
100%
90%
80%
70%
60%
50%
40%
30%
20%
10%
0%
Jun 16 Left
Dec 16 Left
Jun 16 Right
Dec 16 Right
Jun 15 Left
Jun 16 Left
Dec 16 Left
Jun 16 Right
Dec 16 Right
Unstained
Black stains
Acremonium *
Arthroderma
Ascotricha
Aspergillus
Candida
Capronia
Chrysosporium
Clonostachys
Cylindrocarpon
Davidiella
Exophiala *
Fusarium
Geosmithia
Golovinomyces
Gymnoascus
Ilyonectria
Kazachstania
Kurtzmanomyces
Lecanicillium
Leptosphaerulina
Leucosporidiella
Mollisia
Mortierella
Myxotrichum
Naumovozyma
Ochroconis *
Oidiodendron
Penicillium
Phoma
Pochonia
Pseudogymnoascus
Pulchromyces
Purpureocillium
Rhodotorula
Scutellospora
Sporothrix
Stachybotrys
Trichosporon
Xenochalara
Ascomycota_unidentified
Atheliaceae_unidentified
Cordycipitaceae_unidentified
Fungi_unidentified
Glomeraceae_unidentified
Glomeromycetes_unidentified
Herpotrichiellaceae_unidentified *
Hypocreaceae_unidentified
Hypocreales_unidentified
Microascaceae_unidentified
Nectriaceae_unidentified
Pleosporales_unidentified
Sebacinaceae_unidentified
Sordariaceae_unidentified
Sordariomycetes_unidentified
Tremellales_unidentified
Trichocomaceae_unidentified
Bacterial genera
B
n = 6
1
6
2
5
4
2
4
3
100%
90%
80%
70%
60%
50%
40%
30%
20%
10%
0%
Jun 16 Left
Dec 16 Left
Jun 16 Right
Dec 16 Right
Jun 15 Left
Jun 16 Left
Dec 16 Left
Jun 16 Right
Dec 16 Right
Unstained
Black stains
Achromobacter
Acidothermus
Actinophytocola
Advenella
Aeromicrobium
Agromyces
Amaricoccus
Aminobacter
Amycolatopsis
Angiococcus
Bacillus
Bdellovibrio
Blastocatella
Bosea
Bradyrhizobium
Brevundimonas
Bryobacter
Candidatus Alysiosphaera
Candidatus Metachlamydia
Candidatus Protochlamydia
Catelliglobosispora
Chitinophaga
Coxiella
Devosia
Dokdonella
Ferruginibacter
Flavihumibacter
Gemmata
Haliangium
Hyphomicrobium
Inquilinus
Isosphaera
Jiangella
Kribbella
Labrys
Legionella
Lentzea
Luteimonas
Lysinimonas
Mesorhizobium
Methylobacterium
Microbacterium
Mycobacterium
Nannocystis
Neochlamydia
Niabella
Nitrosospira
Nocardia
Nocardioides
Nordella
Novosphingobium
Parapedobacter
Patulibacter
Pedobacter
Pedomicrobium
Phaselicystis
Phenylobacterium
Phreatobacter
Phyllobacterium
Phytomonospora
Pir4 lineage
Planomonospora
Polymorphobacter
Promicromonospora
Propionibacterium
Pseudomonas
Pseudonocardia
Pseudosphingobacterium
Ralstonia
Reyranella
Rhizobium
Rhodococcus
Rhodopseudomonas
Roseomonas
Sandaracinobacter
Sandaracinus
Singulisphaera
Solimonas
Sorangium
Sphingobacterium
Sphingobium
Sphingomonas
Sphingopyxis
Sporichthya
Stenotrophomonas
Steroidobacter
Streptomyces
Tahibacter
Taibaiella
Variibacter
Woodsholea
Zymomonas
Others

Fig. S3

Fig. S4 Community composition at genus level for fungi in black stains and nearby unstained parts sampled from the left and right walls of Lascaux's Apse in June 2015 ('Jun 15'), June 2016 ('Jun 16') and December 2016 ('Dec 16'). Black fungi (i.e. *Ochroconis*) are shown in black and taxa containing both pigmented and non-pigmented strains in dark grey (Herpotrichiellaceae) or light grey (*Exophiala*). Taxa representing more than 1% of sequences are indicated. Each histogram is the average from 1-6 samples (indicated in each case).

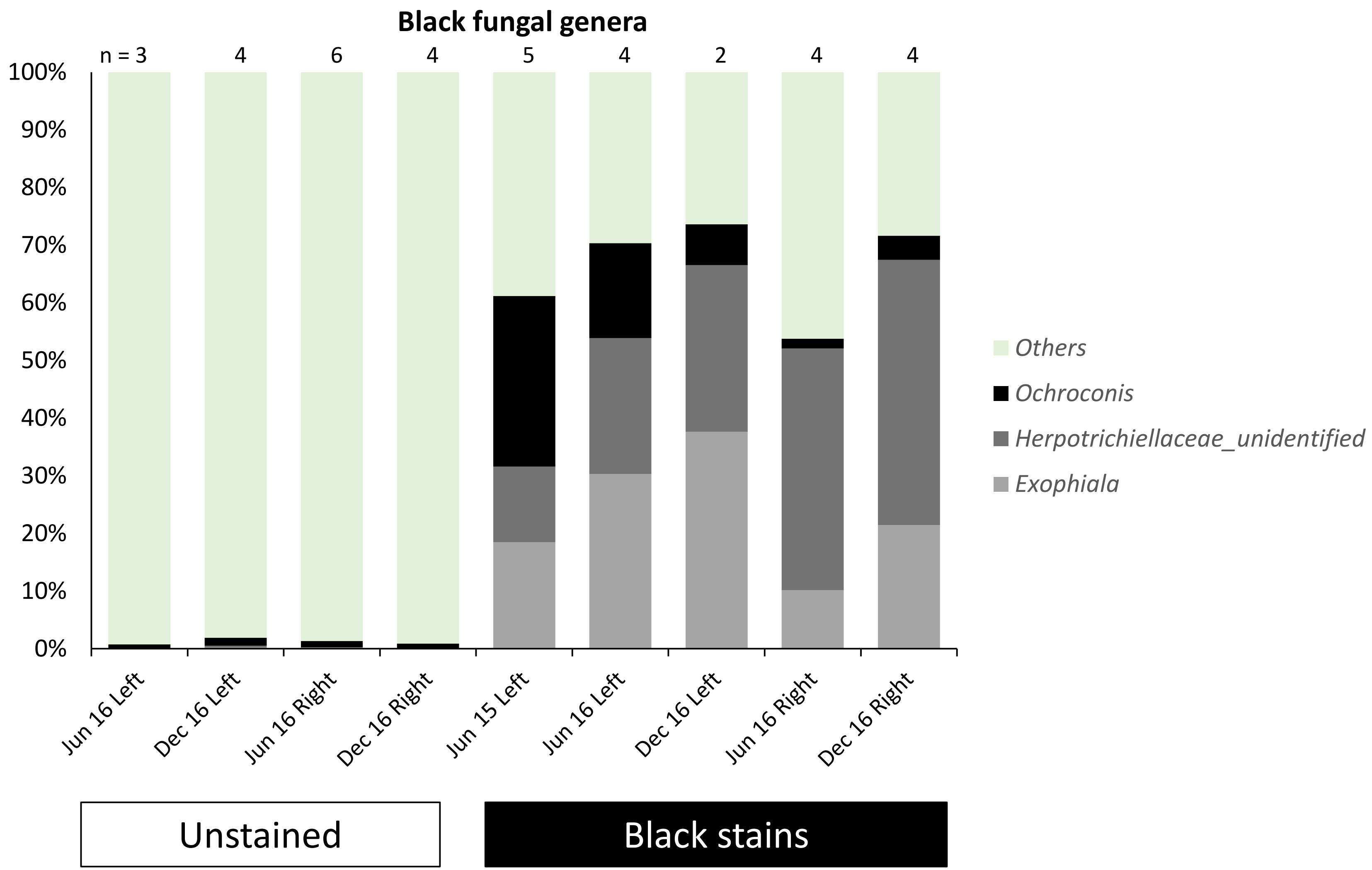

Fig. S4

Fig. S5 Community composition at genus level for micro-eukaryotes in black stains and nearby unstained parts sampled from the left and right walls of Lascaux's Apse in June 2015 ('Jun 15'), June 2016 ('Jun 16') and December 2016 ('Dec 16'). Genera representing more than 1% of sequences are indicated. Each histogram is the average from 2-6 samples (indicated in each case).

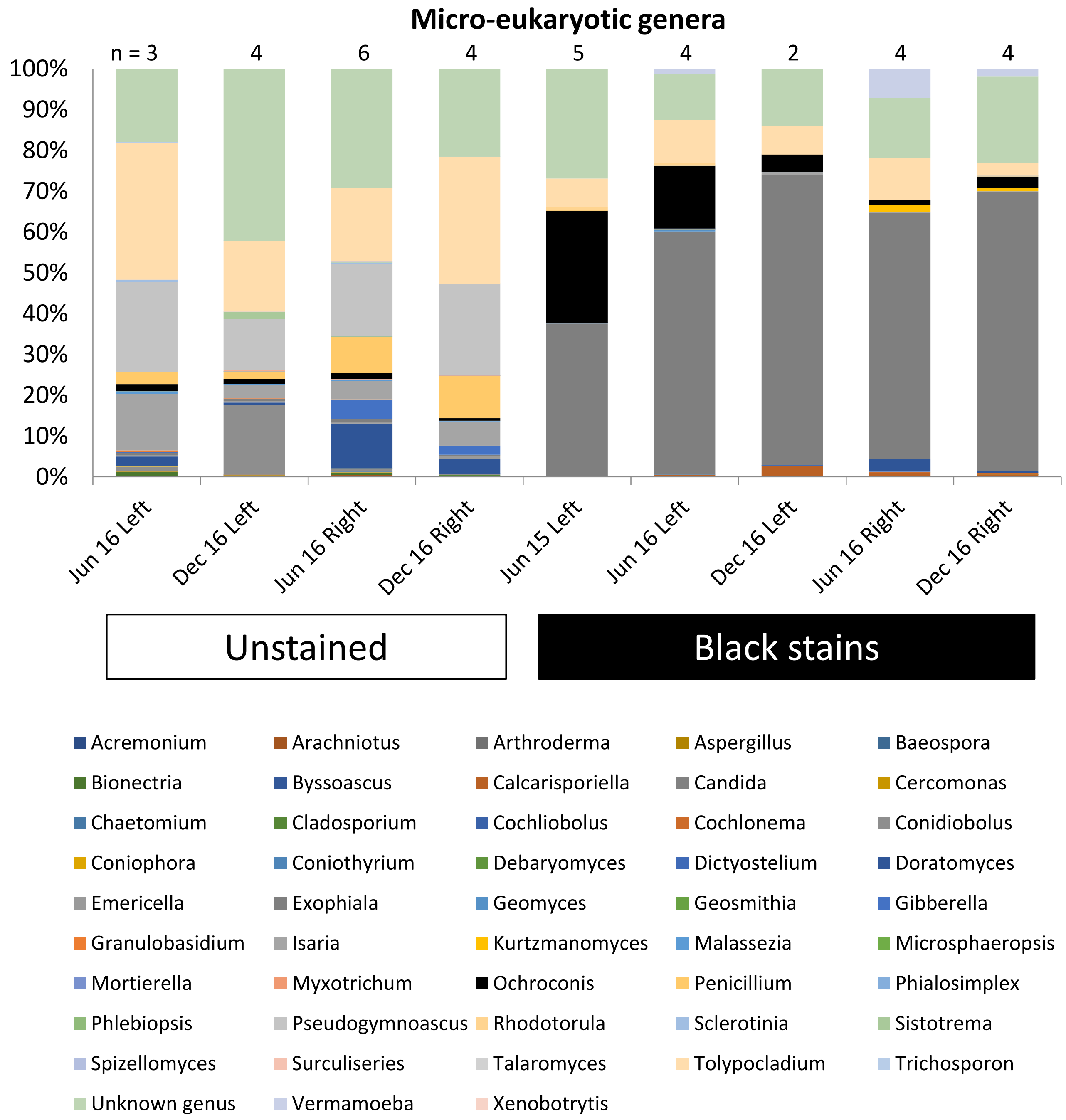

Fig. S5

Fig. S6 Abundance of the bacterial and fungal communities colonizing cave walls in the Apse. The values shown are means with their standard errors. There was no statistical difference between black stains and unstained parts (Wilcoxon tests, $P > 0.05$). qPCR was done using 16S rRNA primers 519F/907R [Laiz et al. 2003], 18S rRNA primers EUK345F/EUK499R [Zhu et al. 2005] and a LightCycler 480 (Roche Diagnostics, Meylan, France). Briefly, the 16S rRNA and 18S rRNA genes reactions were carried out in 20-µl volumes containing 0.6 µl (final concentration 0.3 µM) of each primer, 4 µl of PCR-grade water, 10 µl of LightCycler-DNA Master SYBR Green I master mix (Roche Applied Science, Meylan, France) and 2 µl of sample DNA (5 ng). PCR was done with 10 min at 95°C, followed by 40 cycles of (i) 95°C for 15 s, (ii) 63°C for 60 s, and (iii) 72°C for 30 s for 16S rRNA genes, and 2 min at 95°C, followed by 40 cycles of (i) 95°C for 15 s, (ii) 60°C for 15 s, and (iii) 72°C for 15 s for 18S rRNA genes. Melting curve calculation and Tm determination were done using the Tm Calling Analysis module of Light-Cycler Software v.1.5 (Roche Applied Science).

Laiz L, Piñar G, Lubitz W, Saiz-Jimenez C. Monitoring the colonization of monuments by bacteria: cultivation versus molecular methods. Environ Microbiol. 2003; 5:72-74.
Zhu F, Massana R, Not F, Marie D, Vaulot D. Mapping of picoeucaryotes in marine ecosystems with quantitative PCR of the 18S rRNA gene. FEMS Microbiol Ecol. 2005; 52:79-92.

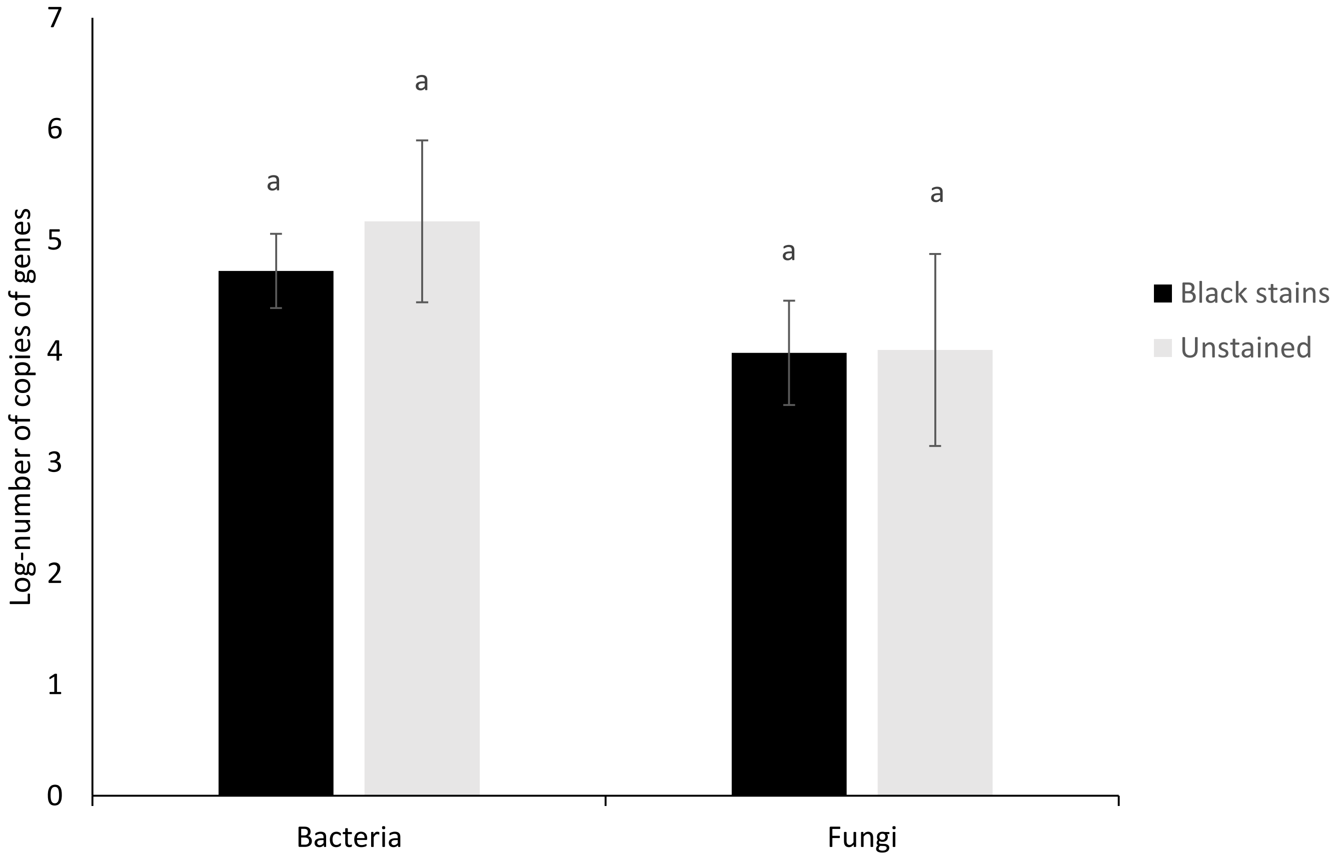

Fig. S6

Fig. S7 Number of OTU (Taxa), Chao1 index of OTU richness (Chao1), Simpson's index of diversity (Simpson 1-D) and Shannon index of OTU diversity (Shannon H') for bacteria, micro-eukaryotes and fungi in Lascaux's Apse walls according to sampling time i.e. June 2015 ('Jun_15'), June 2016 ('Jun_16') and December 2016 ('Dec_16') of the left ('Left') and right ('Right') walls for black stains and nearby unstained parts ('U'). Differences between conditions are shown with lowercase letters (based on ANOVA and Tukey's tests; $P < 0.05$).

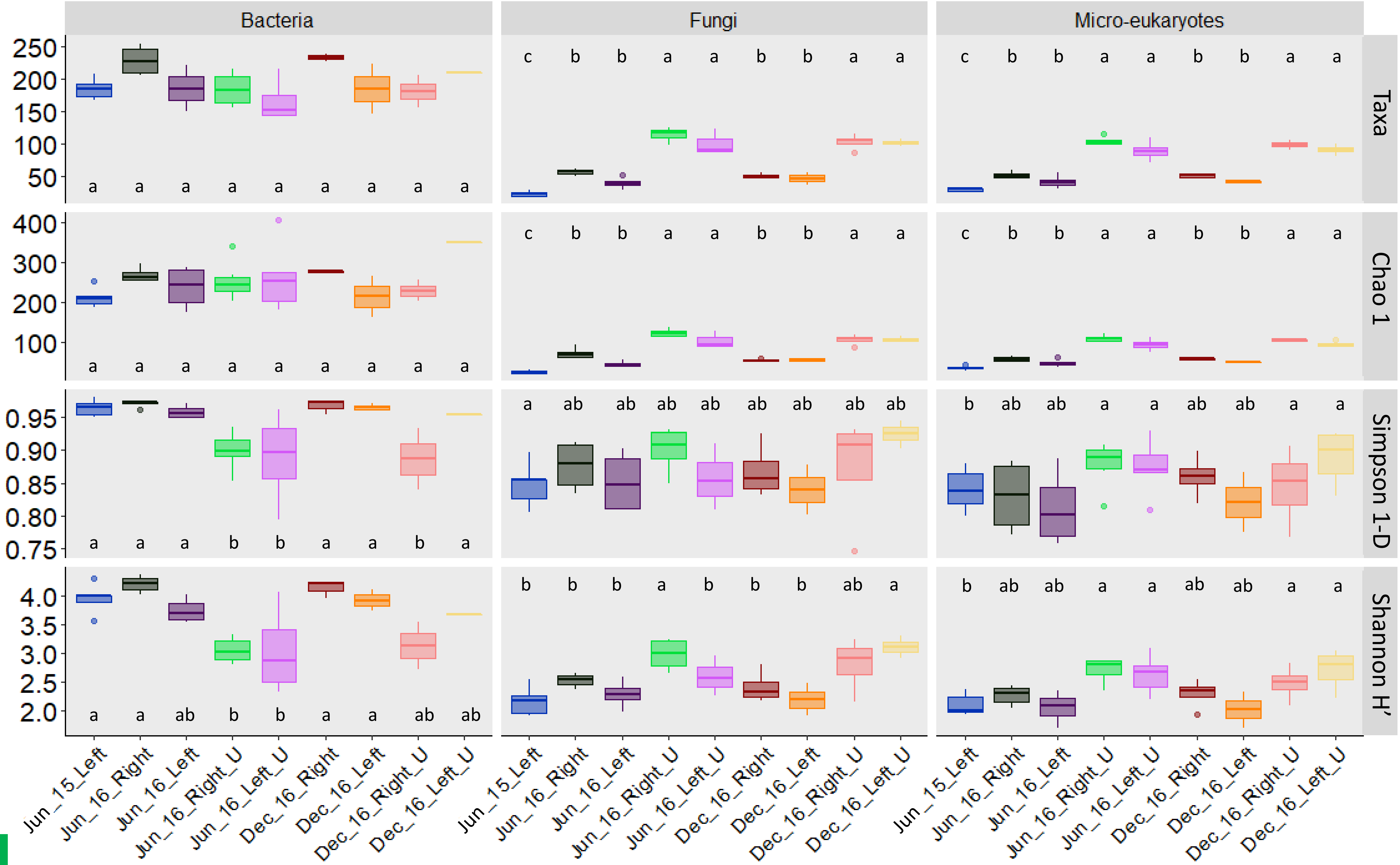

Bacteria
Fungi
Micro-eukaryotes
Taxa
Chao 1
Simpson 1-D
Shannon H'
Jun_15_Left
Jun_16_Right
Jun_16_Left
Jun_16_Right_U
Jun_16_Left_U
Dec_16_Right
Dec_16_Left
Dec_16_Right_U
Dec_16_Left_U

Fig. S7

Fig. S8 Community composition at genus level for non-*Pseudomonas* bacteria in black stains and nearby unstained parts sampled from the left ('Left') and right ('Right') walls of Lascaux's Apse in June 2015 ('Jun 15'), June 2016 ('Jun 16') and December 2016 ('Dec 16'). Genera representing more than 1% of sequences, once sequences of the predominant genus *Pseudomonas* were removed, are indicated. Each histogram is the average from 1-6 samples (indicated in each case). The genus taxonomic profile of bacteria in black stains differed somewhat between cave walls, with e.g. a higher proportion of *Neochlamydia* in right walls than left walls (3.2-6.2% vs 0-1.2%), but otherwise differences between various black stains from a same wall or different sampling dates for a same black stain were minor, i.e. not consistent or typically < 1.5%.

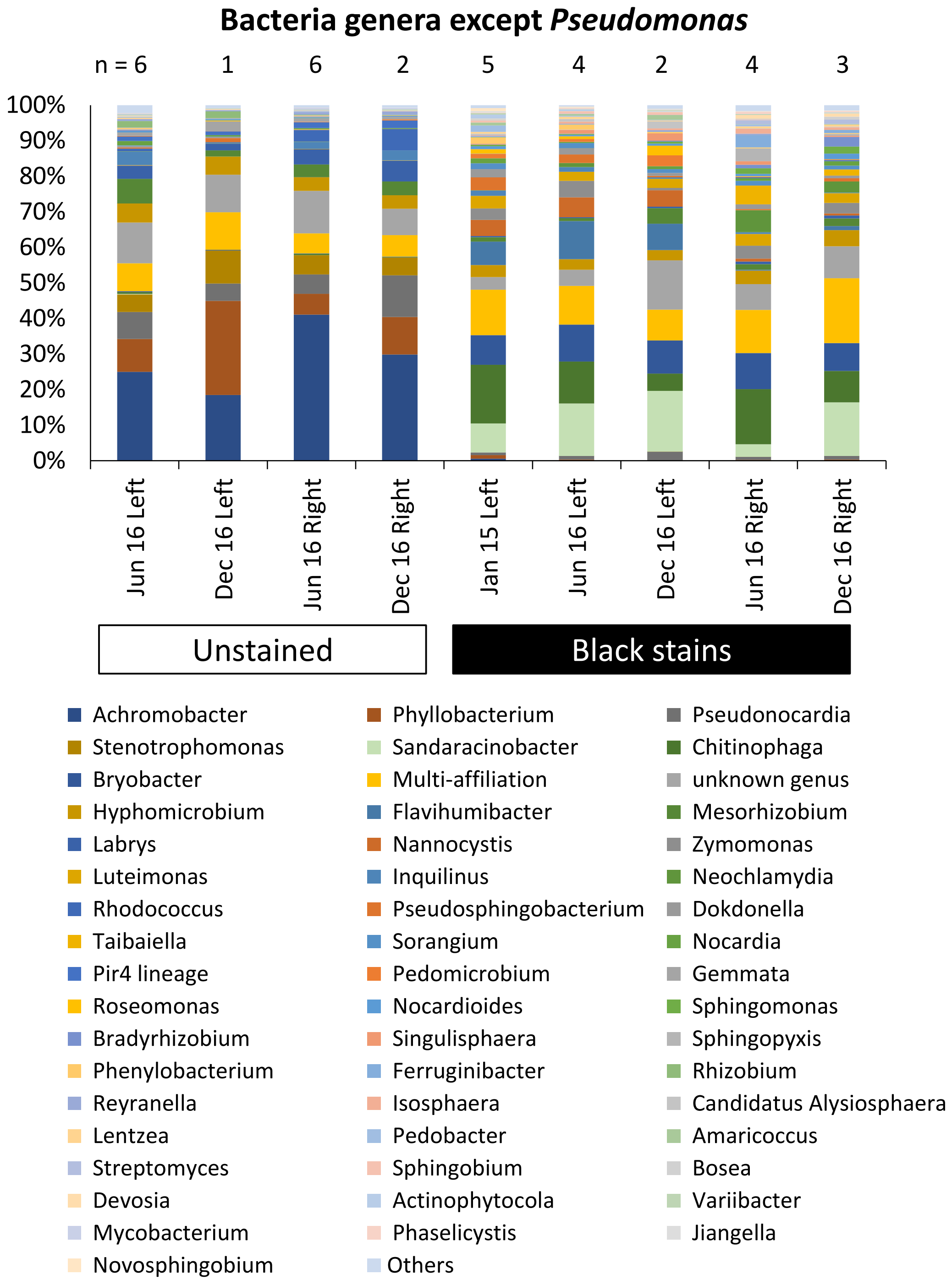

Fig. S8

Fig. S9 Set-up of dual confrontation experiments between *Pseudomonas* isolates (all *Pseudomonas* available from unstained surface belong to clade C2, whereas the black stain *Pseudomonas* isolate is from clade C5) from the Apse and black fungi on plates. Many of the black fungi tested become black at later stages of fungal growth, as illustrated here with *Alternaria alternata* that starts forming a white colony (shown with inhibition effects of *Pseudomonas* at the top right and at the bottom). Top left: the fungus is introduced in the plate as a square plug from which the mycelium develops and forms a large colony.

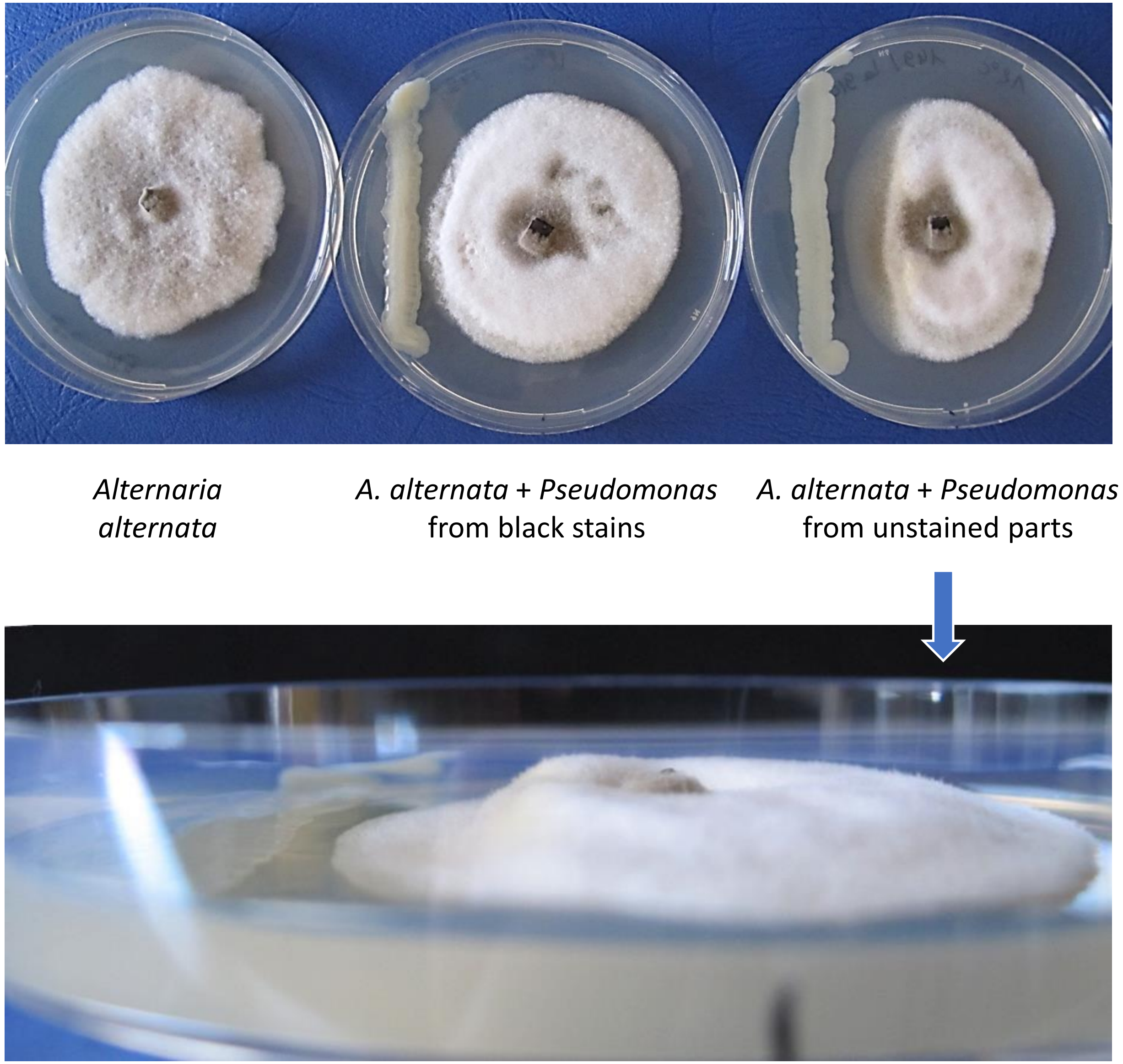

Fig. S9

Fig. S10 Venn Diagram showing unique and shared microbial genera evidenced in collembola from black stains, black stains and unstained parts.
A, Bacterial community (16S rRNA genes). **B**, Fungal community (ITS2 regions).

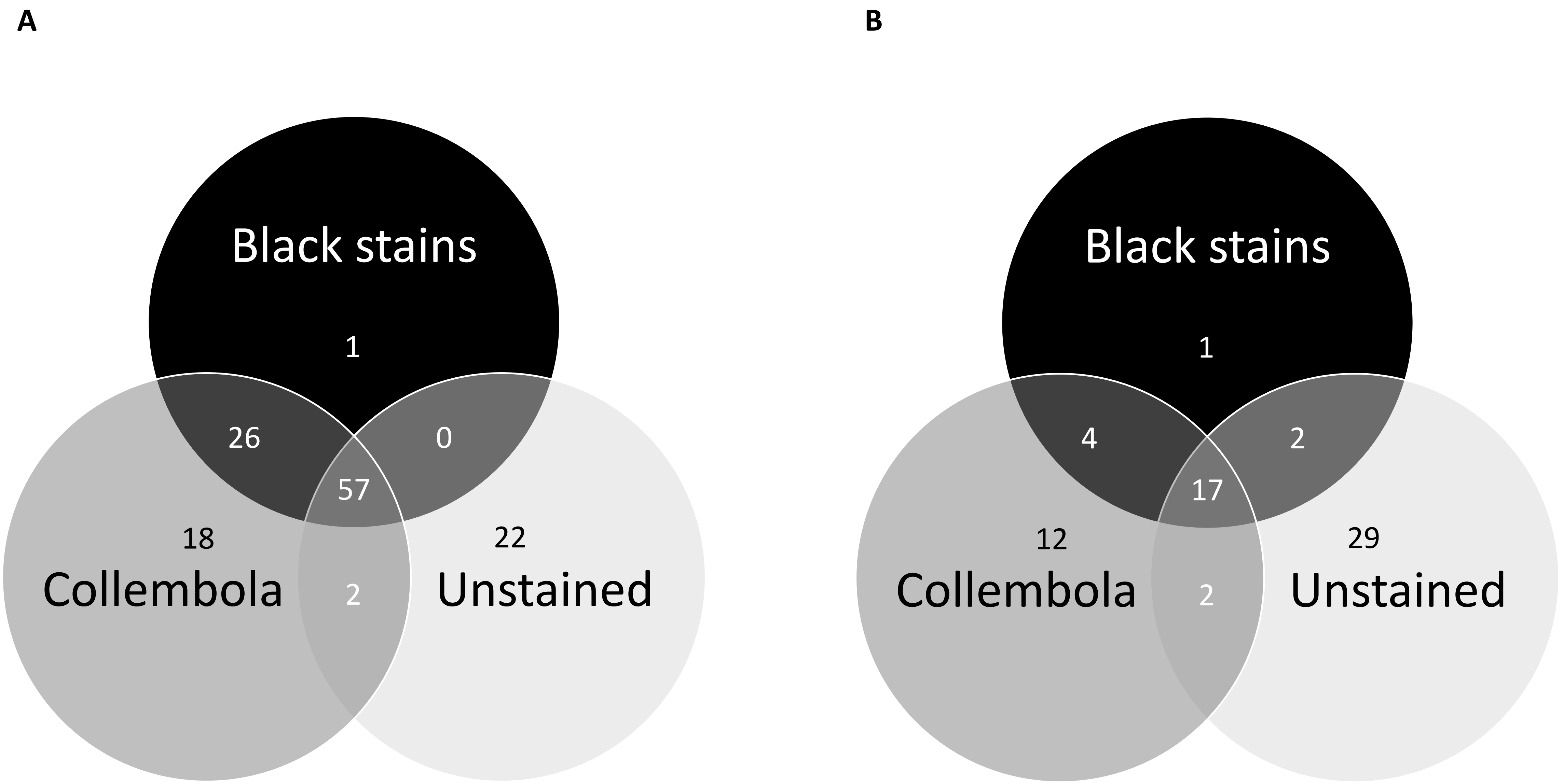

Fig. S10
